# Pauli blocking of stimulated emission in a degenerate Fermi gas

**DOI:** 10.1038/s41467-022-34135-6

**Published:** 2022-10-29

**Authors:** Raphael Jannin, Yuri van der Werf, Kees Steinebach, Hendrick L. Bethlem, Kjeld S. E. Eikema

**Affiliations:** LaserLab, Department of Physics and Astronomy, Vrije Universiteit, De Boelelaan 1081, 1081 HV Amsterdam, The Netherlands

**Keywords:** Ultracold gases, Atomic and molecular interactions with photons

## Abstract

The Pauli exclusion principle in quantum mechanics has a profound influence on the structure of matter and on interactions between fermions. Almost 30 years ago it was predicted that the Pauli exclusion principle could lead to a suppression of spontaneous emission, and only recently several experiments confirmed this phenomenon. Here we report that this so-called Pauli blockade not only affects incoherent processes but also, more generally, coherently driven systems. It manifests itself as an intriguing sub-Doppler narrowing of a doubly-forbidden transition profile in an optically trapped Fermi gas of ^3^He. By actively pumping atoms out of the excited state, we break the coherence of the excitation and lift the narrowing effect, confirming the influence of Pauli blockade on the transition profile. This insight into the interplay between quantum statistics and coherent driving is a promising development for future applications involving fermionic systems.

## Abstract (web summary)

Pauli exclusion principle has fundamental and practical consequences to the structure of matter and particle interaction. Here the authors demonstrate Pauli blocking in a coherently driven system using trapped 3He degenerate Fermi gases.

## Introduction

The Pauli exclusion principle arises from the simple requirement of antisymmetrization of two-fermion wavefunctions under particle exchange^1^, but it has a remarkable impact on how our world is shaped. It determines the electronic structure of atoms, which dictates their chemical properties, and it gives rise to the Fermi energy of electron ensembles in solid state materials, leading to electrical (semi-)conductivity. Another example is the Fermi pressure which stabilizes the densest observable matter in our universe like white dwarves and neutron stars against gravitational collapse. In the field of ultracold atomic physics, the Pauli exclusion principle has a direct impact on the collisional properties of identical fermions, due to the absence of *s*-wave (even parity) collisions, which has been observed experimentally^2,3^, and has cleared the way towards unprecedented fractional uncertainties at or below the 10^−18^ level of state-of-the-art fermionic 1D lattice clocks^4–8^.

Since the idea was suggested almost three decades ago that spontaneous decay of an excited ultracold fermion confined in a Fermi sea would be suppressed due to quantum statistics, it has regularly attracted theoretical interest^9–16^. This suppression of spontaneous emission can become relevant in photon scattering events, where the absorption and subsequent spontaneous emission of a photon imparts a momentum transfer *ℏ***k** = *ℏ*(**k**_abs_ − **k**_emi_) on the atom. If the imparted photon recoil is smaller than the Fermi momentum of the Fermi sea, these scattering events couple to states that are already occupied, and are thus strongly suppressed. This so-called Pauli blockade of spontaneous emission in ultracold degenerate Fermi gases has only recently been experimentally observed^17–19^.

In this work, in contrast, we demonstrate Pauli blockade of stimulated emission in a degenerate Fermi gas of ^3^He. This is shown through the intriguing phenomenon of narrowing the linewidth of the 2^3^S_1_ → 2^1^S_0_ transition, that is studied in our experiment, below what may be expected based on the temperature of the gas. This transition at 1557 nm connects the two metastable states of helium. Due to the very small Einstein coefficient of about 9 × 10^−8^ s^−1^, the upper state lifetime fully determines the natural linewidth of 8 Hz, making this an ideal candidate for precision spectroscopy in helium. Moreover, it is trivial to make the coherent driving Rabi frequency (2π × 40 Hz for our experimental conditions) orders of magnitude larger than the Einstein coefficient, eliminating all influence of spontaneous decay back to the 2^3^S_1_ state. In order to verify the influence of Pauli blockade on the spectral linewidth, we suppress the stimulated emission back to the 2^3^S_1_ state using a “depumper” laser, by which we retrieve Doppler-broadened profiles.

## Results

### Description of the narrowing mechanism

**Fig. 1:**
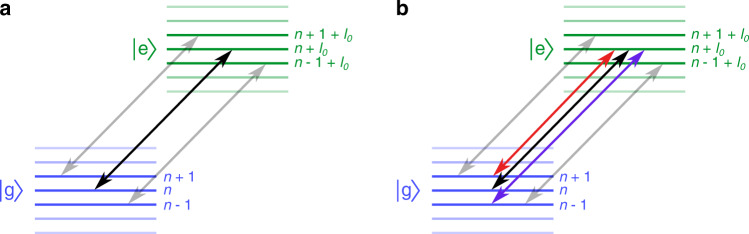
Couplings between the magic wavelength optical dipole trap motional states, induced by the excitation light. **a** When the excitation light contains a single frequency, only pairs of motional states with a fixed energy difference (equal to *ℓ*_0_ vibrational quanta) are coupled (carrier transitions, figured as black and gray arrows). **b** When the spectrum of the excitation laser is broader than the energy splitting of the vibrational states, several other transitions are possible too (sideband transitions, represented as red and blue arrows).

The excitation is performed in a degenerate Fermi gas of ^3^He in the 2^3^S_1_ state, spin-polarized to the *m*_*F*_ = +3/2 Zeeman substate, and confined in a crossed optical dipole trap (ODT) at the magic wavelength (where the trapping potential is identical for both 2^3^S_1_ and 2^1^S_0_ states). Even though the excitation creates a mixture of the triplet and singlet states, the effect of the interactions on the evolution of the gas is considered negligible since for typical samples, *k*_F_*a*_ts_ stays below 0.02 (*ℏk*_F_ representing the Fermi momentum and *a*_ts_ the triplet-singlet *s*-wave scattering length based on that of ^4^He^20^). If the linewidth of the excitation source is narrow enough to resolve the energy difference between the motional states induced by the trapping potential, only pairs of states with the same energy difference are coupled, as illustrated in Fig. 1a. We define this as carrier transitions, and the hole left in the lower state by excitation to the upper state can only be refilled by stimulated emission of the same atom back to the lower state again. The evolution of the system can be described as an ensemble of independent atoms each performing its own Rabi oscillation. The absorption profile will therefore simply reflect the momentum distribution of the atoms in the trap, in the form of a Doppler broadening.

If, on the other hand, the linewidth of the excitation laser is much broader than the energy spacing of the motional levels (which is the case in our experiment), each vibrational state couples to multiple others. This situation is depicted in Fig. 1b. The sideband transitions lead to an exchange of motional states, which is affected by the Pauli exclusion principle. Upon absorption of a photon, de-excitation is again only possible towards states which are not occupied, but now the laser bandwidth covers many more states. The excitation profile will then reflect both the absorption profile, determined by the phase space density, and stimulated emission downward again, which depends on the distribution of holes in the Fermi gas. It ultimately leads to a narrowing of the measured transition below the Doppler width expected based on the temperature of the sample. Counter-intuitively, this happens only if the laser is broad enough to couple different motional states. The model explaining this narrowing effect will be developed in the next paragraphs.

### Observation and modeling of the effect

We can describe the excitation process by the following Hamiltonian in the interaction picture:

1 $$H=\mathop{\sum}\limits_{nm}{a}_{m-n}\frac{{{{\Omega }}}_{{g}_{n},{e}_{m}}}{2}\big({\hat{e}}_{m}^{{{{\dagger}}} }{\hat{g}}_{n}+{\hat{g}}_{n}^{{{{\dagger}}} }{\hat{e}}_{m}\big)\equiv \mathop{\sum}\limits_{nm}{H}_{{g}_{n}{e}_{m}}\big({\hat{e}}_{m}^{{{{\dagger}}} }{\hat{g}}_{n}+{\hat{g}}_{n}^{{{{\dagger}}} }{\hat{e}}_{m}\big),$$

where ${\hat{g}}_{n}^{{{{\dagger}}} }$ and ${\hat{e}}_{m}^{{{{\dagger}}} }$ (${\hat{g}}_{n}$ and ${\hat{e}}_{m}$) represent the creation (annihilation) operators of a fermion in state $\left|{{{{{{{\rm{g}}}}}}}},n\right\rangle$ and $\left|{{{{{{{\rm{e}}}}}}}},m\right\rangle$ respectively, where *n* and *m* represent the motional quantum states for atoms in the lower ($\left|{{{{{{{\rm{g}}}}}}}}\right\rangle$) and upper ($\left|{{{{{{{\rm{e}}}}}}}}\right\rangle$) internal atomic states (see Fig. 1), and ${{{\Omega }}}_{{g}_{n},{e}_{m}}$ represents the Rabi frequencies coupling these states^21^ (see the Supplementary Note 1 for more details). The weights *a*_*m*−*n*_ in Eq. (1) describe the spectral intensity distribution of the excitation laser light, which is peaked at ∣*m* − *n*∣ = *ℓ*_0_. When e.g., only ${a}_{{\ell }_{0}}$ is non-zero, then only carrier transitions are driven, as shown in Fig. 1a. In order to capture the physics resulting from the excitation light, we compute the transition rates ${{{\Gamma }}}_{{{{{{{{{\rm{g}}}}}}}}}_{n}\to {{{{{{{{\rm{e}}}}}}}}}_{m}}$ and ${{{\Gamma }}}_{{{{{{{{{\rm{e}}}}}}}}}_{m}\to {{{{{{{{\rm{g}}}}}}}}}_{n}}$ using Fermi’s golden rule (see the Supplementary Note 1 for details):

2 $${{{\Gamma }}}_{{{{{{{{{\rm{g}}}}}}}}}_{n}\to {{{{{{{{\rm{e}}}}}}}}}_{m}}=\frac{2\pi }{{\hslash }^{2}{\omega }_{{{{{{{{\rm{g}}}}}}}}}}{\left|{H}_{{{{{{{{{\rm{g}}}}}}}}}_{n}{{{{{{{{\rm{e}}}}}}}}}_{m}}\right|}^{2}{n}_{{{{{{{{{\rm{g}}}}}}}}}_{n}}\big(1-{n}_{{{{{{{{{\rm{e}}}}}}}}}_{m}}\big)\,{{{{{{{\rm{and}}}}}}}}$$

3 $${{{\Gamma }}}_{{{{{{{{{\rm{e}}}}}}}}}_{m}\to {{{{{{{{\rm{g}}}}}}}}}_{n}}=\frac{2\pi }{{\hslash }^{2}{\omega }_{{{{{{{{\rm{e}}}}}}}}}}{\left|{H}_{{{{{{{{{\rm{g}}}}}}}}}_{n}{{{{{{{{\rm{e}}}}}}}}}_{m}}\right|}^{2}{n}_{{{{{{{{{\rm{e}}}}}}}}}_{m}}\big(1-{n}_{{{{{{{{{\rm{g}}}}}}}}}_{n}}\big),$$

with ${n}_{{{{{{{{{\rm{g}}}}}}}}}_{n}}$ and ${n}_{{{{{{{{{\rm{e}}}}}}}}}_{m}}$ representing the occupation numbers of the different states and *ω*_g_ (*ω*_e_) corresponding to the trapping frequency felt by the internal state $\left|{{{{{{{\rm{g}}}}}}}}\right\rangle$ ($\left|{{{{{{{\rm{e}}}}}}}}\right\rangle$) (for a magic wavelength ODT, *ω*_g_ = *ω*_e_). These excitation rates reflect the Pauli exclusion principle since they both depend on the occupation of the initial state and the availability of holes in the final state. From this we obtain the shape of our spectroscopy signal (measured as depletion of the 2^3^S_1_ population):

4 $${{{{{{{\mathcal{S}}}}}}}}\propto \mathop{\sum}\limits_{nm}{\left|{a}_{m-n}\right|}^{2}{\left|{{{\Omega }}}_{{{{{{{{{\rm{g}}}}}}}}}_{n}{{{{{{{{\rm{e}}}}}}}}}_{m}}\right|}^{2}\left({n}_{{{{{{{{{\rm{g}}}}}}}}}_{n}}(0)-\frac{{\sum }_{k}{\left|{a}_{m-k}\right|}^{2}{\left|{\tilde{{{\Omega }}}}_{{{{{{{{{\rm{g}}}}}}}}}_{k}{{{{{{{{\rm{e}}}}}}}}}_{m}}\right|}^{2}{n}_{{{{{{{{{\rm{g}}}}}}}}}_{k}}(0)\left(1-{n}_{{{{{{{{{\rm{g}}}}}}}}}_{n}}(0)\right)}{{\sum }_{k}{\left|{a}_{m-k}\right|}^{2}{\left|{\tilde{{{\Omega }}}}_{{{{{{{{{\rm{g}}}}}}}}}_{k}{{{{{{{{\rm{e}}}}}}}}}_{m}}\right|}^{2}+{f}_{{{{{{{{\rm{g}}}}}}}}}{{{\Gamma }}}_{0}/{{{\Omega }}}^{2}}\right),$$

in which 1/Γ_0_ represents the lifetime of the $\left|{{{{{{{\rm{e}}}}}}}}\right\rangle$ state, *f*_g_ = *ω*_g_/2*π* and ${\tilde{{{\Omega }}}}_{{{{{{{{{\rm{g}}}}}}}}}_{k}{{{{{{{{\rm{e}}}}}}}}}_{m}}={{{\Omega }}}_{{{{{{{{{\rm{g}}}}}}}}}_{k}{{{{{{{{\rm{e}}}}}}}}}_{m}}/{{\Omega }}$ (see the Supplementary Note 1 for an elaborate derivation). The first term describes the contribution of absorption to the signal and the second one accounts for exchange of motional states within the $\left|{{{{{{{\rm{g}}}}}}}}\right\rangle$ manifold through stimulated emission, which is subject to Pauli blocking.

To get to a practically applicable model, the motional states are treated in a semi-classical approach and denoted with $\left|{{{{{{{\bf{r}}}}}}}},{{{{{{{\bf{k}}}}}}}}\right\rangle$, where **r** represents the position and *ℏ***k** the momentum of the atoms. The number density of the initial Fermi gas is given by the Fermi–Dirac distribution function^22,23^:

5 $${\rho }_{{{{{{{{\rm{g}}}}}}}}}({{{{{{{\bf{r}}}}}}}},{{{{{{{\bf{k}}}}}}}})=\frac{1}{{(2\pi )}^{3}}\frac{1}{1+\exp \big(\beta {H}_{{{{{{{{\rm{g}}}}}}}}}({{{{{{{\bf{r}}}}}}}},{{{{{{{\bf{k}}}}}}}})-\beta \mu \big)},$$

with *β* = 1/*k*_B_*T*, where *T* and *μ* are the temperature and chemical potential of the gas respectively, and *H*_g_(**r**, **k**) is the Hamiltonian describing state $\left|g\right\rangle$.

Similarly to refs. 12, 14, we use a local density approach to estimate the effective excitation rate between $\left|{{{{{\rm{g}}}}}}\right\rangle$ and $\left|{{{{{\rm{e}}}}}}\right\rangle$. Expression (4) yields (see the Supplementary Note 2 for details):

6 $${{{{{{{\mathcal{S}}}}}}}}(\omega )\propto {{{{{{{{\mathcal{S}}}}}}}}}_{{{{{{{{\rm{Absorption}}}}}}}}}(\omega )\left(1-{{{{{{{\mathcal{M}}}}}}}}(\omega )\right),$$

with

7 $${{{{{{{{\mathcal{S}}}}}}}}}_{{{{{{{{\rm{Absorption}}}}}}}}}(\omega )\propto \int {{{{{{{{\rm{d}}}}}}}}}^{3}{{{{{{{\bf{r}}}}}}}}\int {{{{{{{{\rm{d}}}}}}}}}^{3}{{{{{{{\bf{k}}}}}}}}\,{\rho }_{{{{{{{{\rm{g}}}}}}}}}({{{{{{{\bf{r}}}}}}}},{{{{{{{\bf{k}}}}}}}})\delta (\omega -{\omega }_{{{{{{{{\bf{r}}}}}}}},{{{{{{{\bf{k}}}}}}}}}),$$

and a modification factor ${{{{{{{\mathcal{M}}}}}}}}(\omega )$, representing the Pauli-blocked stimulated emission, defined as:

8 $${{{{{{{\mathcal{M}}}}}}}}(\omega )=\frac{\int {{{{{{{{\rm{d}}}}}}}}}^{3}{{{{{{{\bf{r}}}}}}}}\int {{{{{{{{\rm{d}}}}}}}}}^{3}{{{{{{{\bf{k}}}}}}}}\,{\rho }_{{{{{{{{\rm{g}}}}}}}}}({{{{{{{\bf{r}}}}}}}},{{{{{{{\bf{k}}}}}}}})\big(1-{\rho }_{{{{{{{{\rm{g}}}}}}}}}({{{{{{{\bf{r}}}}}}}},{{{{{{{\bf{k}}}}}}}})\big)\delta (\omega -{\omega }_{{{{{{{{\bf{r}}}}}}}},{{{{{{{\bf{k}}}}}}}}})}{\int {{{{{{{{\rm{d}}}}}}}}}^{3}{{{{{{{\bf{r}}}}}}}}\int {{{{{{{{\rm{d}}}}}}}}}^{3}{{{{{{{\bf{k}}}}}}}}\,{\rho }_{{{{{{{{\rm{g}}}}}}}}}({{{{{{{\bf{r}}}}}}}},{{{{{{{\bf{k}}}}}}}})\delta (\omega -{\omega }_{{{{{{{{\bf{r}}}}}}}},{{{{{{{\bf{k}}}}}}}}})}.$$

In the Supplementary Note 2, a full evaluation of Eqs. (6)–(8) is given. In principle ${{{{{{{\mathcal{M}}}}}}}}(\omega )$ depends also on the spectral profile of the excitation laser, but to include this effect would significantly complicate the calculation. As the linewidth of the excitation laser is much broader than the energy spacing of the motional states, we therefore only consider that the main contribution will come from the center frequency and thus neglect the small change in position or momentum.

**Fig. 2:**
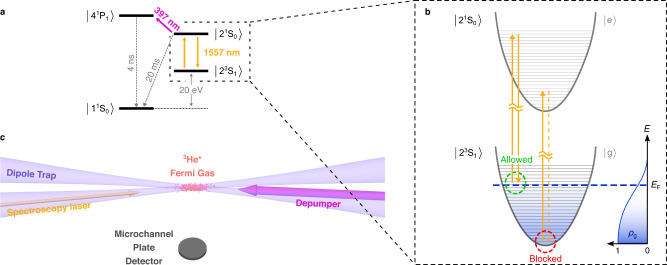
Description of the experimental configuration and the Pauli blocking mechanism. **a** Energy scheme showing the relevant electronic levels of helium. A depumper between the 2^1^S_0_ and the 4^1^P_1_ states can artificially reduce the lifetime of the 2^1^S_0_ state. **b** Vibrational states induced by the optical dipole potential and depiction of the Pauli blocking mechanism depending on the state occupation: stimulated emission is inhibited for low-energy states whereas it is allowed for high-energy states. This results in a narrowing of the spectrum. **c** Schematic of the experiment. The Fermi gas is trapped in a crossed dipole trap at 320 nm. The spectroscopy beam at 1557 nm counterpropagates with respect to one of the beams from the ODT. A depumper at 397 nm is sent on the atomic cloud along its long axis. After excitation, the remaining part of the DFG is released from the ODT and is detected by a microchannel plate detector located below the trapping region.

Figure 2a shows the energy levels of helium that are relevant to our experiment. A graphical representation of the influence of the ODT induced potentials and of ${{{{{{{\mathcal{M}}}}}}}}(\omega )$ on the 2^3^S_1_ → 2^1^S_0_ excitation process is also shown in Fig. 2b, illustrating the narrowing mechanism. Atoms with (low) momentum *p* ≪ *p*_F_ (*p*_F_ representing the Fermi momentum) are excited at the center of the spectral profile, corresponding to atoms populating the low energy states of the confining potential. For those atoms almost no holes are available in a degenerate Fermi gas to go back to, so ${{{{{{{\mathcal{M}}}}}}}}(\omega )$ goes to zero and stimulated emission is suppressed. On the other hand, atoms with *p* ≃ *p*_F_ are excited at the wings of the spectral profile due to the Doppler effect, for these atoms the availability of holes goes to unity. Stimulated emission back is allowed, leading to a reduced effective excitation rate from $\left|{{{{{{{\rm{g}}}}}}}}\right\rangle$ to $\left|{{{{{{{\rm{e}}}}}}}}\right\rangle$. As the Doppler effect is the only broadening mechanism for fermions in a magic wavelength ODT, the spectral linewidth narrows compared to pure Doppler broadening. Moreover, the effect does not induce any additional shift on the center frequency since it is fully symmetrical in momentum space. The validity of Eq. (6) describing this phenomenon was confirmed by also performing a few-body simulation based on numerically solving the master equation describing the dynamics of the system (see the Supplementary Note 3).

Most of the experimental apparatus has been described in earlier works^20,24,25^. To produce a degenerate Fermi gas (DFG) of ^3^He in the metastable state 2^3^S_1_(*F* = 3/2, *m*_*F*_ = +3/2) we first perform sympathetic cooling along with bosonic ^4^He. The mixture is evaporatively cooled down to quantum degeneracy in a cloverleaf magnetic trap until only a pure Fermi gas of ^3^He is left. The gas is then transferred to a crossed optical dipole trap (ODT) at the magic wavelength for the 2^3^S_1_ → 2^1^S_0_ transition at 319.8 nm^26,27^, with typical confining frequencies of *ω*_//_ = 2*π* × 15 Hz in the axial direction and *ω*_⊥_ = 2π × 120 Hz in the radial ones. We typically produce Fermi gases composed of a few 10^5^ to few 10^6^ atoms at temperatures *T*/*T*_F_ ranging from 1.5 to 0.25. The excitation to the 2^1^S_0_(*F* = 1/2, *m*_*F*_ = +1/2) is then performed using a laser beam at 1557 nm, with a spectral linewidth of about 5 kHz^20^, counter-propagating with respect to the incident beam of the ODT, corresponding to the axial direction of the trap, as shown in Fig. 2c. After 3 s of excitation, the metastable helium cloud is released from the trap and falls under gravity onto a micro-channel plate detector (MCP) producing a time-of-flight signal. This provides a way to measure the number of atoms of the gas, its temperature and chemical potential. Because of the relatively short lifetime of about 20 ms of the 2^1^S_0_ state compared to the time of free-fall, only atoms left in the 2^3^S_1_ state are detected, and excitation to the singlet metastable state is translated into a reduction of the number of trapped atoms. This is our spectroscopic signal, and each realization of the experimental sequence is followed by one where a measurement of the DFG atom number without excitation light is made to probe the fluctuations of the atom number over time.

**Fig. 3:**
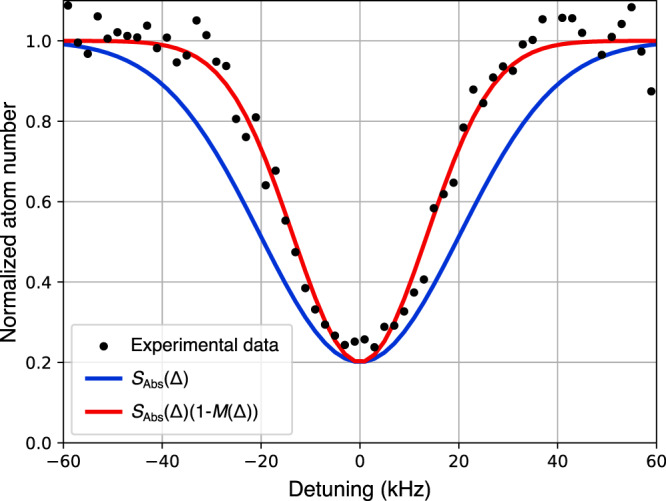
Comparison of the observed spectrum to the models. The measured spectrum (black dots) is plotted together with the calculated Doppler broadened profile (solid blue line) and the narrowed profile based on Pauli-blocking when no depumper light is present (solid red line). Each data point is followed by a background shot for which no probe light is present, and a polynomial fit to the temporal evolution of the background is used for the normalization of the shown data. We did not add errorbars to the data points as they would only reflect the accuracy with which the number of atoms can be determined from a time-of-flight profile, but not be representative of the statistics of the experiment, which can be observed as the 10 to 15% scatter between individual points.

In Fig. 3, our experimental observation of Pauli blocking on stimulated emission is shown for the 2^3^S_1_ → 2^1^S_0_ transition at a temperature of *T*/*T*_F_ ≃ 0.55. Compared to the calculated Doppler width (the blue line in Fig. 3), the transition is narrowed by a factor 0.75, clearly indicating the expected effect of Pauli blocking. Note that the observed linewidth is independent of the excitation time and amount of depletion, which was confirmed by simulations. This is related to the low Rabi frequencies involved in the excitation and the absence of rethermalizing *s*-wave collisions in the ultracold Fermi gas.

### Test of the model through cancellation

From Eq. (4), for ${{{{{{{\mathcal{M}}}}}}}}(\omega )$ to modify the line profile significantly, the lifetime *τ* = 1/Γ_0_ of the $\left|{{{{{{{\rm{e}}}}}}}}\right\rangle$ internal state should be longer than *f*_g_/Ω^2^. Therefore, the Pauli blockade effect can be suppressed by artificially decreasing the lifetime of state $\left|{{{{{{{\rm{e}}}}}}}}\right\rangle$. This prevents excited atoms to be stimulated back to the $\left|{{{{{{{\rm{g}}}}}}}}\right\rangle$ state, so that a Doppler broadened profile is retrieved. It effectively creates the same situation as previously demonstrated for helium in a 1557 nm ODT^28^ (blue-detuned dipole potential for the 2^1^S_0_ state), where the excited state $\left|{{{{{{{\rm{e}}}}}}}}\right\rangle$ is expelled from the trap so that it cannot be stimulated back. Artificially decreasing the lifetime of the upper state thus provides an experimental way to verify the influence of Pauli blockade on stimulated emission.

We can effectively decrease the lifetime of the 2^1^S_0_ state by exciting it to the short-lived 4^1^P_1_ state with a 397 nm “depumper” laser^29,30^. The relevant four-level system is depicted in Fig. 2a. Any excitation to the 4^1^P_1_ state will result in a quick decay to the 1^1^S_0_ level since the Einstein A coefficient for this transition is 35 times higher than the one leading back to 2^1^S_0_^31^. Therefore, those atoms will be lost from the Fermi gas. By varying the intensity of the 397 nm depumper, we are able to adjust the effective lifetime of the 2^1^S_0_ state (1/Γ_0_ in Eq. (4)) and thus change the effect of the modification factor ${{{{{{{\mathcal{M}}}}}}}}(\omega )$ on the linewidth.

**Fig. 4:**
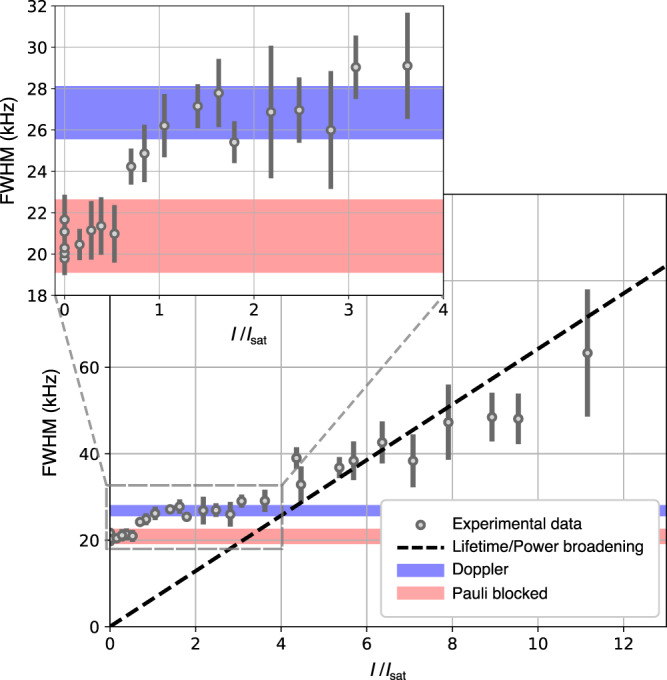
Dependence of the FWHM on the depumper intensity. The observed FWHM (gray dots) is shown as a function of depumper intensity expressed in terms of the saturation intensity as defined in the text and the Supplementary Note 5. The errorbars on these points represent the error on the FWHM obtained from fitting Gaussian profiles to the measured line profiles. The regimes where narrowing happens as a consequence of Pauli blocking and where the profile is fully Doppler-dominated are seen as the first and second plateau respectively. Their expected values from the spread of the parameters of the Fermi gases are displayed as red and blue areas respectively. The dashed black line shows the expected FWHM based on only lifetime and power broadening obtained from numerically solving the optical Bloch equations describing the evolution of a four level system for a single atom. The inset provides a zoom of the Pauli blocking and Doppler broadened regime.

Figure 4 shows the observed full width at half maximum (FWHM) of the spectroscopic lines as a function of depumper laser intensity *I*. The two shaded regions indicate the expected linewidths, for the two mentioned regimes, calculated with expression (6) based on averaged thermodynamical values of the Fermi gases (see the “Methods” section). In order to isolate any trivial broadening due to the depumper (lifetime or power broadening), we numerically solve the optical Bloch equations averaged over the Fermi–Dirac distribution of the gas for the level scheme shown in Fig. 2a (see the “Methods” section for details), from which we extract an expected FWHM and we define an effective saturation intensity *I*_sat_ of ~ 15 μW/cm^2^ (details of this evaluation are given in the Supplementary Note 5). The predicted FWHM obtained using this procedure as a function of depumper intensity is shown as a dashed line. At intensities above approximately 4*I*_sat_ (60 μW/cm^2^), the width is dominated by single-atom lifetime broadening due to the depumper beam. Below that intensity, the quantum statistical effects can be distinguished in two clear regimes. The first one appears when no depumper light (or a small intensity) is applied. This case corresponds to the first plateau (FWHM ~ 21 kHz) seen in Fig. 4, with a sub-Doppler linewidth due to Pauli blockade.

As the intensity of the depumper beam is increased, the lifetime of the singlet state becomes shorter so that eventually stimulated emission is suppressed at saturation (when *I* becomes comparable to *I*_sat_), and therefore the influence of Pauli blockade disappears. This regime constitutes the second plateau (FWHM ~ 27 kHz) seen in Fig. 4. The expected values of the two plateaus are well predicted by expression (6) considering an effective lifetime for the excited state. In order to also predict the threshold intensity where the transition happens between the two regimes, one would have to include the full effect of the excitation laser linewidth in the model. As mentioned before, this would further complicate the calculations significantly, and was therefore not pursued.

### Dependence on temperature

**Fig. 5:**
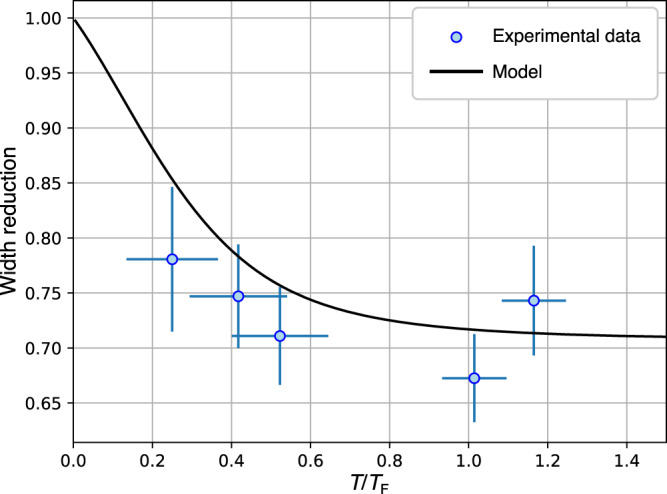
Reduction of the FWHM due to the effect of Pauli blocking of stimulated emission. The FWHM is plotted as a function of the temperature *T*/*T*_F_ as determined before each experiment. The solid black curve corresponds to the ratio of the expected widths of ${{{{{{{\mathcal{S}}}}}}}}(\omega )$ calculated using expression (6), and ${{{{{{{{\mathcal{S}}}}}}}}}_{{{{{{{{\rm{Absorption}}}}}}}}}(\omega )$. Blue points represent the values of the ratio of the two plateaus averaged over intervals of *T*/*T*_F_ values. Error bars are obtained by binning the data based on their value of *T*/*T*_F_.

By varying our experimental cooling and trapping conditions, we can change the parameters of the produced Fermi gases over a range of temperatures and chemical potentials. It is then possible to construct a universal curve displaying the linewidth reduction factor as a function of temperature. This is shown in Fig. 5. In the limit of zero temperature, all states below the Fermi energy are occupied (except one hole from excitation), hence it is very improbable for excited atoms to be stimulated back into the lower electronic state, resulting in a Doppler excitation profile. As the temperature of the Fermi gas increases, the availability of holes around the Fermi energy increases too and stimulated emission becomes possible, leading to an increased narrowing of the line profile. When *T* ≫ *T*_F_, the quantum statistical nature of the atoms does not play a role anymore in the expression of the rates (2) and (3), and the Doppler profile should be retrieved again (this is out of the validity range of the presented theoretical approach). As can be seen in Fig. 5, the reduction of the actual FWHM compared to the Doppler linewidth becomes stronger as *T*/*T*_F_ increases until it reaches a reduction by a factor ~ 0.7 when the temperature is comparable to the Fermi temperature. Although it was not possible to achieve temperatures lower than *T* ≃ 0.25*T*_F_ to measure into the deeply degenerate regime where the availability of holes becomes negligible, the experimental values consistently show a significant narrowing compared to the Doppler width, with a good agreement to the theoretical curve over the full range of experimental parameters.

## Discussion

In contrast to what was observed by^17–19^, our study shows that Pauli blockade can affect coherent processes in specific situations where quantum exchange symmetry plays a role. Interestingly, a broader linewidth of the excitation laser results in this case in a narrowing of the spectroscopic linewidth. Even though we are experimentally limited to temperatures down to *T*/*T*_F_ ≃ 0.25, our narrowed spectra mostly reflect the low momentum atoms, so we nevertheless achieve linewidths which are otherwise only reachable for highly degenerate gases. In a broader context, our observation of Pauli blockade of coherent processes could be of interest in the context of cooling of fermionic samples. Even though cooling schemes based on the suppression of spontaneous emission^32^ could allow to achieve degeneracies comparable to the state-of-the-art, the technique is limited by the long time needed for efficient cooling at low *T*/*T*_F_ during which heating is induced by the trapping light. This limitation could be overcome by making use of the blockade of stimulated emission instead, which would provide more tunability, but a cooling scheme has not been designed for it yet. Additionally, the mechanism shows similarities with conduction phenomena in semi-conductor materials and could be used to perform quantum simulations of such materials, or be of great interest for the growing field of quantum logic and information processing with fermionic species^33^ by removing the constraint to map the fermionic degrees of freedom into qubits^34,35^.

In conclusion, we observed a signature of Pauli blockade in a coherently driven system, by the means of spectroscopy of a doubly-forbidden transition in an ultracold Fermi gas of metastable ^3^He. Due to a dependence of this Pauli blockade effect on the laser detuning, we observe reduced spectroscopic linewidths compared to the expected Doppler profiles arising from the finite momentum distribution of the gas. We show that, when actively reducing the lifetime of the excited state, we prevent all stimulated emission events and eliminate the Pauli blockade effect, by which the Doppler width is retrieved. The measured data are in excellent agreement over a range of parameters with the expectations from the model, which only requires the input of thermodynamic and laser parameters. In the context of precision spectroscopy, it proves to be a useful feature as it helps improving the accuracy of the determination of the transition frequency without inducing any additional shift. In a broader perspective, the narrowing effect showcases Pauli blockade of stimulated emission. This influence of quantum statistics on a coherently driven system shows great potential as a tool for applications with many-body physics and quantum information.

## Methods

### Experimental sequence

A beam of ^4^He and ^3^He in the metastable 2^3^S_1_ state is produced using a DC discharge. The beam of atoms is collimated before entering a 2 m long Zeeman slower and subsequently captured by a 3D magneto-optical trap (MOT), producing samples at temperatures around 600 μK. The MOT is loaded with a mixture of the two isotopes for 5 s. The atomic cloud is then transferred to a cloverleaf magnetic trap where it is polarized to the *m*_*J*_ = +1 (^4^He) or the *m*_*F*_ = +3/2 (^3^He) spin states. The obtained spin-stretched cloud is then 1D-Doppler cooled for 3 s, which lowers the temperature to about 110 μK. Forced evaporative cooling is then performed on the mixture of the two isotopes by ramping down the frequency of an RF-knife from 50 to 3 MHz in 3 s. Collisions between the two isotopes allow rethermalization of the ^3^He component through sympathetic cooling. At the final RF frequency, depending on the value of the offset magnetic field produced by the trap coils, all ^4^He has evaporated and only a Fermi gas of ^3^He is left in the magnetic trap. The magic wavelength ODT beams, which are generated by doubling 640 nm light produced by sum-frequency mixing of two beams at 1557 nm and 1086 nm, are then turned on before the confining magnetic field is turned off adiabatically in 50 ms. Detection of the cloud is performed by turning off the ODT beams and free fall of the atoms onto an MCP located 17.5 cm below the trapping region. A time-of-flight profile is fitted to the recorded trace from which the thermodynamical parameters (*N*, *μ,* and *T*) are extracted.

### Data acquisition and analysis

Before and after each measurement of a spectroscopic profile, a set of reference Fermi gases is measured in order to extract thermodynamical parameters (*N*, *μ,* and *T*). These two sets are averaged in order to account for any drift or noise during the realization of the spectroscopy scan. Each spectroscopic measurement is obtained by holding a DFG in the ODT for 3 s while it is exposed to excitation light, after which the same sequence is performed again with the probe light turned off in order to account for atom number fluctuations. A depletion of up to 80% of the initial trapped atoms is achieved at the resonance frequency. While the spectroscopy laser is on, a constant magnetic field of 4 G is applied to minimize depolarization of the sample. A spectrum is then built up by determining the number of atoms left in the gas for each laser frequency, normalized to a polynomial fit of the reference measurements where no light was present. For typical conditions, the spectral line shape we obtain can be well approximated and fitted with a Gaussian profile, from which we determine the FWHM of the line. The degeneracy of the initial Fermi gas can be varied by a combination of changing the sweeping rate of the RF knife frequency and changing the power of the ODT beams. The data shown in Fig. 4 have been acquired using 300 mW of light in each beam of the crossed ODT and a trapped Fermi gas composed of about 1.5 × 10^5^ atoms at a temperature of ~100 nK. These data are combined in Fig. 5 with other sets of data, obtained with DFGs composed of 10^5^ to 10^6^ atoms and temperatures ranging from 100 to 300 nK. Displayed as a function of depumper laser intensity, these data exhibit a similar behavior showing two regimes as those in Fig. 4 (see Supplementary Note 6 and Supplementary Fig. 4). For each set, we determine the temperature *T*/*T*_F_ through the relation:

9 $$\frac{T}{{T}_{{{{{{{{\rm{F}}}}}}}}}}={\left(-6{{{{{{{{\rm{Li}}}}}}}}}_{3}(-\zeta )\right)}^{-1/3},$$

where Li_3_(−*ζ*) is the trilogarithm function and *ζ* = e^*βμ*^ is the fugacity of the gas. The measurements are divided into two categories: below the depumping intensity threshold where the stimulated emission is relevant, and above this threshold where the stimulated emission is eliminated. We discard lifetime-broadened measurements from the analysis shown in Fig. 5. The two sets of data obtained this way are then binned based on their values of *T*/*T*_F_ and a value of the linewidth is obtained by averaging over the range of the binning interval. Finally, the ratio between the widths of the profiles, with and without the contribution of the Pauli blocked stimulated emission, is calculated and errors are propagated.

### Generation of the spectroscopy light

We here give a brief overview of the spectroscopy laser infrastructure. More details can be found in refs. 20, 28. The spectroscopy light is generated using an erbium fiber laser (NKT Photonics E15) at 1557 nm, which is transfer-locked to an ultrastable erbium fiber laser (ORS1500 from Menlo Systems) at 1542 nm, through an optical frequency comb (OFC) from Menlo Systems with a center wavelength at 1500 nm and a bandwidth of 100 nm. The ultrastable laser is locked to a reference cavity by the Pound-Drever-Hall technique, such that its specified linewidth is less than 1 Hz with a stability of 10^−15^ at 1 s. Two beat notes (one for each of the two lasers) with the OFC are generated before being mixed together to generate a virtual beat note which is finally mixed to the signal generated by a caesium clock (Symmetricom CsIII Model 4310B) in order to produce the error signal used to lock the spectroscopy laser. The control over its frequency, needed to perform the precision spectroscopy measurements, is achieved by mixing the beat note signal produced with the OFC with the signal generated by a direct digital synthesizer (DDS). Estimations of the different contributions to the linewidth of the spectroscopy laser have been performed (see ref. 36) and summarize as ~1 kHz due to the OFC, ~2 kHz due to the phase noise introduced by the 80 m long fiber linking the OFC laboratory to the experimental setup, and ~1–2 kHz due to the noise induced by the electronics used for the lock of the laser. This last contribution has been measured by performing a delayed self-heterodyne beat note measurement using a 100 km fiber link. Based on these contributions, the total laser linewidth is estimated to be ~5 kHz, which was later found to be consistent with the measurements performed in ref. 28.

### Generation of the depumper light

The light resonant with the 2^1^S_0_ → 4^1^P_1_ transition at 396.603 nm is generated by an external cavity laser diode locked to a saturated absorption signal from a discharge cell of ^3^He. The frequency is also continuously monitored using a High Finesse WS-U 30 wavelength meter to prevent any drift during the rather long (~1 h) acquisition of a spectroscopic scan.

### Estimation of the lifetime broadening

Lifetime broadening due to the coupling of the 2^1^S_0_ and the 4^1^P_1_ states is estimated by solving a set of 16 optical Bloch equations (accounting for the 4 populations and 12 coherences of the 4 internal states) describing the dynamics of the 1^1^S_0_, 2^3^S_1_, 2^1^S_0_ and 4^1^P_1_ as shown on Fig. 2a (see for example ref. 37). All values of the Einstein coefficients are taken from ref. 31. Such a set of equations can be written in the form:

10 $$\dot{{{{{{{{\boldsymbol{\rho }}}}}}}}}={{{{{{{\bf{M}}}}}}}}\cdot {{{{{{{\boldsymbol{\rho }}}}}}}},$$

where the density matrix **ρ** is flattened into a 16 elements column vector and **M** is a 16 × 16 matrix containing the couplings. The solution is obtained by propagating the initial density matrix by numerically computing the expression:

11 $${{{{{{{\boldsymbol{\rho }}}}}}}}(t)={{{{{{\rm{e}}}}}}}^{{{{{{{{\bf{M}}}}}}}}t}\cdot {{{{{{{\boldsymbol{\rho }}}}}}}}(0),$$

where initially the only non-zero element is the one describing a population in the metastable triplet state (equal to 1). The 2^3^S_1_ population at time *t* = 3 s is evaluated over a range of 500 kHz around the resonance frequency to build up a spectrum, from which the FWHM is extracted. Since our modeling describes only single atom dynamics, the coupling between the metastable triplet and singlet states inserted in Eq. (11) is obtained by averaging the Rabi frequency over the Fermi–Dirac distribution following a similar approach as in ref. 38 (see the Supplementary Note 4).

## Supplementary information

Supplementary Information

## Data Availability

The data and codes that support the plots in this study are provided in the Supplementary Source Data file. The raw data generated in this study are available from the corresponding author upon request. Source data are provided with this paper.

## Source data

Source Data
