# Supplementary material for: Pauli blocking of stimulated emission in a degenerate Fermi gas
Source: Nat Commun. 2022 Oct 29;13:6479. doi: 10.1038/s41467-022-34135-6 (PMC9617905; doi:10.1038/s41467-022-34135-6)
Supplement: Supplementary file 1 — Supplementary Information [file 41467_2022_34135_MOESM1_ESM.pdf]

Supplementary material to
"Pauli blocking of stimulated emission in a degenerate Fermi gas"

R. Jannin[1*], Y. van der Werf[1], K. Steinebach[1], H.L. Bethlem[1], and K.S.E. Eikema[1]

[1]LaserLaB, Department of Physics and Astronomy, Vrije Universiteit, De Boelelaan 1081, 1081 HV Amsterdam, the Netherlands

Supplementary Note 1: Calculation of the transition rates and derivation of the line profile

As explained in the main text, we consider a Fermi gas in an internal state $|\mathrm{g}\rangle$ confined in a harmonic potential. This trapping potential has an angular frequency ω_g along its axial direction. State $|\mathrm{g}\rangle$ is coupled at time $t = 0$ to the excited state $|e\rangle$ by resonant light with a wavenumber κ and angular frequency ω. The energy difference between these two levels is $\hbar\omega_0$. The excitation laser beam propagates along the axial direction of the trapping potential (which we denote $/\!\!/$ whereas the radial direction is denoted $\perp$). The atoms in state $|\mathrm{e}\rangle$ feel a harmonic potential with an axial angular frequency ω_e, which in the particular case of a magic wavelength trap is $\omega_\mathrm{e} = \omega_\mathrm{g}$. The confinement potential is assumed to be weak compared to the recoil energy $\hbar\omega_\mathrm{rec}$ of the transition, such that the system is out of the Lamb-Dicke regime ($\eta \gg 1$, with the Lamb-Dicke parameter defined as $\eta = \sqrt{\omega_\mathrm{rec}/\omega_\mathrm{g}}$ and the recoil energy $\hbar\omega_\mathrm{rec} = \hbar^2\kappa^2/2m$). For the transition of metastable helium considered in this study, the Lamb-Dicke parameter is $\eta \simeq 30$.

Consider first that the excitation light has a narrower spectral linewidth than the splitting in energy of the motional states. It is assumed to be tuned to a central frequency $\omega = \omega_0 + \ell_0\omega_g$. The system can be described by the following Hamiltonian in the interaction picture:

$$H = \sum_n \frac{\hbar\Omega_{n,n+\ell_0}}{2}\left(\hat{e}^\dagger_{n+\ell_0}\hat{g}_n + \hat{e}_{n+\ell_0}\hat{g}^\dagger_n\right), \tag{1}$$

where $\hat{i}^\dagger_n$ and $\hat{i}_n$ represent the fermionic creation and annihilation operators of an atom in internal state $|i\rangle$ and motional state $|n\rangle$, and the Rabi angular frequencies are given by [1]:

$$\Omega_{n,n+\ell_0} = \Omega e^{-\eta^2/2}\eta^{|\ell_0|}\sqrt{\frac{n_<!}{n_>!}}L^{|\ell_0|}_{n_<}(\eta^2), \tag{2}$$

where $n_<$ ($n_>$) is the lesser (greater) of n and $n + \ell_0$. The evolution of the system is straightforward and leads to independent Rabi oscillations between pairs of $|\mathrm{g}, n\rangle$ and $|\mathrm{e}, n + \ell_0\rangle$ states, as depicted on Figure 1(a) from the main text. These sets of transitions constitute the carrier transitions of the system. Since all these transitions couple pairs of states independently, no collective many-body effect is expected and each fermion composing the Fermi sea can be seen as a single particle.

Now consider the case where the excitation laser is broader than the energy splitting due to the confining potential. The previous Hamiltonian is modified and reads:

$$H = \sum_n \sum_{\delta\ell=-\infty}^{+\infty} a_{\delta\ell}(\ell_0)\frac{\hbar\Omega_{n,n+\ell_0+\delta\ell}}{2}\left(\hat{e}^\dagger_{n+\ell_0+\delta\ell}\hat{g}_n + \hat{g}^\dagger_n\hat{e}_{n+\ell_0+\delta\ell}\right), \tag{3}$$

where $a_{\delta\ell}(\ell_0)$ accounts for the spectral density of the excitation laser at frequency $\omega_0 + (\ell_0 + \delta\ell)\omega_g$. We will use in the following the more compact and convenient expression:

$$H = \sum_{nm} H_{\mathrm{g}_n\mathrm{e}_m}\left(\hat{e}^\dagger_m\hat{g}_n + \hat{g}^\dagger_n\hat{e}_m\right), \tag{4}$$

with $H_{\mathrm{g}_n\mathrm{e}_m}/\hbar = a_{m-n}\Omega_{\mathrm{g}_n,\mathrm{e}_m}/2$. From this expression, it is clear that sideband transitions, as shown in Figure 1(b) from the main text, will be driven in addition to carrier transitions. Hence, the Pauli exclusion principle has to be taken into account to model the proper time evolution of the system.

In our experiment, the internal state $|\mathrm{e}\rangle$ ($2^1\mathrm{S}_0$ state) has a lifetime $\tau = 1/\Gamma_0$ of 20 ms before it decays to the ground state ($1^1\mathrm{S}_0$ state) which is not trapped by the dipole potential. Hence, we are only interested in the first-order transition rates between the states $|\mathrm{g}, n\rangle$ and $|\mathrm{e}, m\rangle$. First, we examine the case of the

*r.jannin@vu.nl

$|\mathrm{g}, n\rangle \rightarrow |\mathrm{e}, m\rangle$ transition. In the occupation number representation, the initial state involved in the transition reads $|i\rangle = |n_{\mathrm{g}_n}, n_{\mathrm{e}_m}\rangle$ and the final state is necessarily:

$$|f\rangle = |n_{\mathrm{g}_n} - 1, n_{\mathrm{e}_m} + 1\rangle = \frac{\hat{e}_m^\dagger \hat{g}_n}{\sqrt{n_{\mathrm{g}_n}\left(1 - n_{\mathrm{e}_m}\right)}} |i\rangle. \tag{5}$$

The coupling matrix element then reads:

$$\langle f|H|i\rangle = H_{\mathrm{g}_n\mathrm{e}_m} \frac{\langle i|\hat{g}_n^\dagger \hat{e}_m \hat{e}_m^\dagger \hat{g}_n |i\rangle}{\sqrt{n_{\mathrm{g}_n}\left(1 - n_{\mathrm{e}_m}\right)}} = H_{\mathrm{g}_n\mathrm{e}_m} \sqrt{n_{\mathrm{g}_n}\left(1 - n_{\mathrm{e}_m}\right)}, \tag{6}$$

where we have used the anti-commutation of fermionic operators and from which we get the transition rate using Fermi's golden rule:

$$\Gamma_{\mathrm{g}_n \rightarrow \mathrm{e}_m} = \frac{2\pi}{\hbar^2 \omega_\mathrm{g}} \left|H_{\mathrm{g}_n\mathrm{e}_m}\right|^2 n_{\mathrm{g}_n}\left(1 - n_{\mathrm{e}_m}\right), \tag{7}$$

since the density of states for a harmonic oscillator is $\rho(\epsilon) = 1/\hbar\omega_\mathrm{g}$. A very similar reasoning gives the rate from $|\mathrm{e}, m\rangle$ to $|\mathrm{g}, n\rangle$:

$$\Gamma_{\mathrm{e}_m \rightarrow \mathrm{g}_n} = \frac{2\pi}{\hbar^2 \omega_\mathrm{e}} \left|H_{\mathrm{g}_n\mathrm{e}_m}\right|^2 n_{\mathrm{e}_m}\left(1 - n_{\mathrm{g}_n}\right). \tag{8}$$

These expressions clearly show that the two transitions are Pauli-blocked and that the transition probability will depend on the occupation number of the final state.

In order to model our system, we consider a simple rate equation for the $|e, m\rangle$ state:

$$\dot{n}_{\mathrm{e}_m} = \sum_n \Gamma_{\mathrm{g}_n \rightarrow \mathrm{e}_m} - \sum_n \Gamma_{\mathrm{e}_m \rightarrow \mathrm{g}_n} - \Gamma_0 n_{\mathrm{e}_m}, \tag{9}$$

from which we determine the condition on the occupation number for a steady state to be reached, $\dot{n}_{\mathrm{e}_m} = 0$. Using the expressions for the rates (7) and (8) and transforming the last equation, we obtain:

$$n_{\mathrm{e}_m} = \frac{\omega_\mathrm{e}}{\omega_\mathrm{g}} \frac{\sum_j \left|H_{\mathrm{g}_j\mathrm{e}_m}\right|^2 n_{\mathrm{g}_j}}{\sum_j \left|H_{\mathrm{g}_j\mathrm{e}_m}\right|^2 \left[1 - n_{\mathrm{g}_j}\left(1 - \omega_\mathrm{e}/\omega_\mathrm{g}\right)\right] + \hbar^2 \omega_\mathrm{e} \Gamma_0 / 2\pi}, \tag{10}$$

which, in the limit where $\omega_\mathrm{e}/\omega_\mathrm{g} \simeq 1$ (close to the magic wavelength condition), simplifies to:

$$n_{\mathrm{e}_m} = \frac{\sum_j \left|H_{\mathrm{g}_j\mathrm{e}_m}\right|^2 n_{\mathrm{g}_j}}{\sum_j \left|H_{\mathrm{g}_j\mathrm{e}_m}\right|^2 + \hbar^2 \omega_\mathrm{g} \Gamma_0 / 2\pi}. \tag{11}$$

The occupation number of the state $|\mathrm{g}, n\rangle$ after a small excitation time Δt (compared to the excitation rate) reads:

$$\begin{aligned} n_{\mathrm{g}_n}(\Delta t) \simeq\ & n_{\mathrm{g}_n}(0) - \frac{2\pi}{\hbar^2} \frac{\Delta t}{\omega_\mathrm{g}} \sum_m \left|H_{\mathrm{g}_n\mathrm{e}_m}\right|^2 n_{\mathrm{g}_n}(0) \\ & + \frac{2\pi}{\hbar^2} \frac{\Delta t}{\omega_\mathrm{g}} \sum_m \left|H_{\mathrm{g}_n\mathrm{e}_m}\right|^2 n_{\mathrm{e}_m}(\Delta t)\left(1 - n_{\mathrm{g}_n}(0)\right), \end{aligned} \tag{12}$$

in which we can replace $n_{\mathrm{e}_m}(\Delta t)$ by the expression (11), assuming that Δt is long enough for a steady state to have been reached ($\Delta t \gg 1/\Gamma_0$). We obtain:

$$\begin{aligned} n_{\mathrm{g}_n}(\Delta t) \simeq\ & n_{\mathrm{g}_n}(0) - \frac{2\pi}{\hbar^2} \frac{\Delta t}{\omega_\mathrm{g}} \sum_m \Bigg[\left|H_{\mathrm{g}_n\mathrm{e}_m}\right|^2 n_{\mathrm{g}_n}(0) \\ & - \frac{\sum_j \left|H_{\mathrm{g}_n\mathrm{e}_m}\right|^2 \left|H_{\mathrm{g}_j\mathrm{e}_m}\right|^2 n_{\mathrm{g}_j}(0)\left(1 - n_{\mathrm{g}_n}(0)\right)}{\sum_j \left|H_{\mathrm{g}_j\mathrm{e}_m}\right|^2 + \hbar^2 \omega_\mathrm{g} \Gamma_0 / 2\pi}\Bigg], \end{aligned} \tag{13}$$

from which we get the total population in the internal $|\mathrm{g}\rangle$ state as:

$$n_\mathrm{g}(\Delta t) = \sum_n n_{\mathrm{g}_n}(\Delta t). \tag{14}$$

The last term of expression (13) is similar to a second-order Fermi golden rule but takes its origin in the fermionic nature of the atomic species. It describes an exchange of motional state within the $|\mathrm{g}\rangle$ internal state through a combination of the absorption of a photon followed by a stimulated emission process, which

is Pauli-blocked by the presence of the Fermi sea. From this term, it is clear that if the excitation light only couples the sets of states $|\mathrm{g}, n\rangle$ and $|\mathrm{e}, n+l_0\rangle$, then this term vanishes, the system does not exhibit Pauli blocking anymore and the previous expression reduces to a common Fermi's golden rule.

We then write the shape of the depletion signal as:

$$\mathcal{S}(\omega) \propto \sum_{nm} |a_{m-n}|^2 \left|\Omega_{\mathrm{g}_n\mathrm{e}_m}\right|^2 \left(n_{\mathrm{g}_n}(0) - \frac{\sum_j |a_{m-j}|^2 \left|\tilde{\Omega}_{\mathrm{g}_j\mathrm{e}_m}\right|^2 n_{\mathrm{g}_j}(0)\left(1-n_{\mathrm{g}_n}(0)\right)}{\sum_j |a_{m-j}|^2 \left|\tilde{\Omega}_{\mathrm{g}_j\mathrm{e}_m}\right|^2 + f_\mathrm{g}\Gamma_0/\Omega^2} \right), \tag{15}$$

with $f_\mathrm{g} = \omega_\mathrm{g}/2\pi$ and $\tilde{\Omega}_{\mathrm{g}_j\mathrm{e}_m} = \Omega_{\mathrm{g}_j\mathrm{e}_m}/\Omega$.

Because the strength of the couplings is modulated by the power spectrum of the excitation light, we expect that the main contributions to the last term come from the cases where $|n-j|$ is small. We also note that in the limit where $\Gamma_0 \gg \Omega^2/f_\mathrm{g}$ (which can be obtained by artificially decreasing the lifetime of the $|\mathrm{e}\rangle$ state as explained in the main text), the second term in the last expression vanishes, thus cancelling the effect of the Pauli blockade. In normal operation of our experiment and without pumping to the $4^1\mathrm{P}_1$ state, we have $\tau = 20$ ms, a free Rabi frequency of about 40 Hz and typical trap frequencies of 10 to 30 Hz, corresponding to a parameter $f_\mathrm{g}\Gamma_0/\Omega^2$ ranging from 2.4×10^{-2} to 8×10^{-3}, which can be neglected as assumed in the previous derivation.

Supplementary Note 2: Calculation of the line profile in the Thomas-Fermi approach

Because of the complexity of the dynamics, we restrict ourselves to a local density approach. Semi-classically, we consider the one-body unperturbed Hamiltonians for both internal states, which are given by:

$$H_{\mathrm{g,e}}(\mathbf{r},\mathbf{p}) = \frac{\mathbf{p}^2}{2m} + \frac{m}{2}\omega_{\mathrm{g,e}}^2\left(\alpha_x^2x^2 + \alpha_y^2y^2 + \alpha_z^2z^2\right), \tag{16}$$

where the coefficients α_x, α_y and α_z account for the anisotropy of the trapping potential. The interaction with light is treated as a perturbation which transfers momentum to the atoms, allowing to make use of the Thomas-Fermi approximation. In such an approximation, the density of states of the DFG in state $|\mathrm{g}\rangle$ is given by:

$$\rho_\mathrm{g}(\mathbf{r},\mathbf{k}) = \frac{1}{(2\pi)^3}\frac{1}{1+\exp\left(\beta H_\mathrm{g}(\mathbf{r},\hbar\mathbf{k}) - \beta\mu\right)}, \tag{17}$$

where $\beta = 1/(k_\mathrm{B}T)$ is the reciprocal temperature, and k_B Boltzmann's constant.

The line profile is found by rewriting the expression (15) within the Thomas-Fermi approximation in the same spirit as [2]. We assume for simplicity that the function defined in expression (11) is peaked at $j=n$ when its denominator is not dominated by $f_\mathrm{g}\Gamma_0/\Omega^2$ (thereby neglecting the small change in phase space induced by stimulated emission). We then simplify it as:

$$\mathcal{S}(\omega) \underset{\sim}{\propto} \sum_{nm} |a_{m-n}|^2 \left|\Omega_{\mathrm{g}_n\mathrm{e}_m}\right|^2 \left[n_{\mathrm{g}_n}(0) - n_{\mathrm{g}_n}(0)\left(1-n_{\mathrm{g}_n}(0)\right)\right], \tag{18}$$

such that we can rewrite it in the semiclassical approach as:

$$\mathcal{S}(\omega) \propto \int \mathrm{d}^3\mathbf{r} \int \mathrm{d}^3\mathbf{k}\ \left[\rho_\mathrm{g}(\mathbf{r},\mathbf{k}) - \rho_\mathrm{g}(\mathbf{r},\mathbf{k})\left(1-\rho_\mathrm{g}(\mathbf{r},\mathbf{k})\right)\right]\delta(\omega-\omega_{\mathbf{r},\mathbf{k}}), \tag{19}$$

with the resonance condition

$$\omega_{\mathbf{r},\mathbf{k}} = \omega_0 + \omega_\mathrm{rec} + \frac{2\hbar\kappa k_{/\!/}}{2m} + \frac{m}{2\hbar}\left(\bar{\omega}_\mathrm{e}^2 - \bar{\omega}_\mathrm{g}^2\right)r^2. \tag{20}$$

The lineshape thus reads:

$$\mathcal{S}(\omega) \propto \mathcal{S}_\mathrm{Absorption}(\omega)\left(1-\mathcal{M}(\omega)\right), \tag{21}$$

with the absorption profile given by [2]:

$$\mathcal{S}_\mathrm{Absorption}(\omega) = \int \mathrm{d}^3\mathbf{r} \int \mathrm{d}^3\mathbf{k}\ \rho_\mathrm{g}(\mathbf{r},\mathbf{k})\delta(\omega-\omega_{\mathbf{r},\mathbf{k}}), \tag{22}$$

and the modification factor expressed as:

$$\mathcal{M}(\omega) = \frac{\int \mathrm{d}^3\mathbf{r}\int \mathrm{d}^3\mathbf{k}\ \rho_\mathrm{g}(\mathbf{r},\mathbf{k})\left(1-\rho_\mathrm{g}(\mathbf{r},\mathbf{k})\right)\delta(\omega-\omega_{\mathbf{r},\mathbf{k}})}{\int \mathrm{d}^3\mathbf{r}\int \mathrm{d}^3\mathbf{k}\ \rho_\mathrm{g}(\mathbf{r},\mathbf{k})\delta(\omega-\omega_{\mathbf{r},\mathbf{k}})}. \tag{23}$$

It can be rewritten as:

$$\mathcal{M}(\omega) = \left[1 - \frac{\int \mathrm{d}^3\mathbf{r} \int \mathrm{d}^3\mathbf{k}\ \rho_\mathrm{g}^2(\mathbf{r},\mathbf{k})\delta(\omega - \omega_{\mathbf{r},\mathbf{k}})}{\int \mathrm{d}^3\mathbf{r} \int \mathrm{d}^3\mathbf{k}\ \rho_\mathrm{g}(\mathbf{r},\mathbf{k})\delta(\omega - \omega_{\mathbf{r},\mathbf{k}})}\right], \tag{24}$$

in which the second term represents the Pauli blocking induced inhibition factor on stimulated emission.

We follow the derivation from [2] to obtain an expression for $\mathcal{S}_\mathrm{Absorption}(\omega)$. Making use of the spherical symmetry over space and cylindrical one over momenta, we rewrite the integrals as:

$$\int \mathrm{d}^3\mathbf{r} \int \mathrm{d}^3\mathbf{k} = 8\pi^2 \int_0^\infty r^2 \mathrm{d}r \int_0^\infty k_\perp \mathrm{d}k_\perp \int_{-\infty}^\infty \mathrm{d}k_{/\!/}, \tag{25}$$

which yields after integration over the axial momenta $k_{/\!/}$:

$$\mathcal{S}_\mathrm{Absorption}(\omega) = \frac{m}{\pi\hbar\kappa} \int_0^\infty r^2 \mathrm{d}r \int_0^\infty k_\perp \mathrm{d}k_\perp \frac{1}{1 + \exp\left[\frac{\beta m}{2}\bar{\omega}_\mathrm{g}^2 r^2 + \frac{\beta\hbar}{4\omega_\mathrm{rec}}\tilde{\omega}^2 - \beta\mu + \frac{\beta\hbar^2}{2m}k_\perp^2\right]}, \tag{26}$$

with

$$\tilde{\omega} = \omega - \omega_0 - \omega_\mathrm{rec} - \frac{m}{2\hbar}\left(\bar{\omega}_\mathrm{e}^2 - \bar{\omega}_\mathrm{g}^2\right) r^2. \tag{27}$$

Performing the integration over the transverse momenta $k_\perp$, performing the change of variable $R = r\bar{\omega}_\mathrm{g}\sqrt{\beta m/2}$ and denoting the detuning $\Delta = \omega - \omega_0 - \omega_\mathrm{rec}$, we can obtain an expression for $\mathcal{S}_\mathrm{Absorption}(\Delta)$:

$$\mathcal{S}_\mathrm{Absorption}(\Delta) \propto \int_0^\infty \mathrm{d}R\ R^2\ \ln\left[1 + e^{-F(R,\Delta)}\right], \tag{28}$$

with $F(R,\Delta) = R^2 + \frac{\hbar\beta}{4\omega_\mathrm{rec}}\left(\Delta + \frac{m_\mathrm{ex}}{\beta\hbar}R^2\right)^2 - \beta\mu$, where $m_\mathrm{ex} = 1 - \omega_\mathrm{e}^2/\omega_\mathrm{g}^2$ characterizes the deviation from the magic wavelength case, and where R is only an integration variable.

We follow the exact same procedure for the calculation of $\int \mathrm{d}^3\mathbf{r} \int \mathrm{d}^3\mathbf{k}\ \rho_\mathrm{g}^2(\mathbf{r},\mathbf{k})\delta(\omega - \omega_{\mathbf{r},\mathbf{k}})$, considering the magic wavelength case where $\omega_\mathrm{g} = \omega_\mathrm{e}$ and $m_\mathrm{ex} = 0$, and we find :

$$\mathcal{M}(\Delta) = \frac{1}{\mathcal{S}_\mathrm{Absorption}(\Delta)} \int_0^\infty \mathrm{d}R\ \frac{R^2}{1 + e^{F(R,\Delta)}}, \tag{29}$$

with $F(R,\Delta)$ which now reads:

$$F(R,\Delta) = R^2 + \frac{\hbar\beta}{4\omega_\mathrm{rec}}\Delta^2 - \beta\mu. \tag{30}$$

Integrating $\mathcal{S}_\mathrm{Absorption}(\Delta)$ and $\mathcal{M}(\Delta)$ then gives:

$$\mathcal{S}_\mathrm{Absorption}(\Delta) \propto -\frac{\sqrt{\pi}}{4}\mathrm{Li}_{5/2}\left[-\zeta \exp\left(-\frac{\hbar\beta}{4\omega_\mathrm{rec}}\Delta^2\right)\right], \tag{31}$$

$$\mathcal{M}(\Delta) = \frac{\mathrm{Li}_{3/2}\left[-\zeta \exp\left(-\frac{\hbar\beta}{4\omega_\mathrm{rec}}\Delta^2\right)\right]}{\mathrm{Li}_{5/2}\left[-\zeta \exp\left(-\frac{\hbar\beta}{4\omega_\mathrm{rec}}\Delta^2\right)\right]}, \tag{32}$$

where the fugacity is $\zeta = e^{\beta\mu}$ and $\mathrm{Li}_n(z)$ denotes the n-th order polylogarithm function of z.

Hence, $\mathcal{S}(\Delta)$ becomes:

$$\mathcal{S}(\Delta) \propto \mathrm{Li}_{5/2}\left[-\zeta\ \exp\left(-\frac{\hbar\beta}{4\omega_\mathrm{rec}}\Delta^2\right)\right] - \mathrm{Li}_{3/2}\left[-\zeta\ \exp\left(-\frac{\hbar\beta}{4\omega_\mathrm{rec}}\Delta^2\right)\right]. \tag{33}$$

In case the lifetime of the $|\mathrm{e}\rangle$ internal state τ becomes small compared to f_g/Ω^2 (by applying sufficient intensity in the depump laser from the $2^1\mathrm{S}_0$ to the $4^1\mathrm{P}_1$ states as presented in the main text), the second term related to $\mathcal{M}(\omega)$, vanishes and we retrieve the non Pauli-blocked profile as derived in [2] and experimentally verified in [3] (in that case, the condition $\tau \to 0$ was realized by the fact that the $|\mathrm{e}\rangle$ state was expelled from the dipole trap by a blue-detuned harmonic potential).

Supplementary Note 3: Few-body numerical analysis

In order to verify our modelling, we numerically solved the master equation describing both the coherent excitation and the decoherence due to the decay to the $1^1\mathrm{S}_0$ state. The equation reads:

$$\dot{\rho}(t) = -\frac{i}{\hbar}\left[\hat{H}, \rho(t)\right] + \sum_n \frac{1}{2}\left(2\hat{C}_n\rho(t)\hat{C}_n^\dagger - \rho(t)\hat{C}_n^\dagger\hat{C}_n - \hat{C}_n^\dagger\hat{C}_n\rho(t)\right), \tag{34}$$

where $\rho(t)$ represents the density matrix of the system at time t, and with the Hamiltonian given in expression (3) and the collapse operators defined as:

$$\hat{C}_n^\dagger = \sqrt{\Gamma_0}\ \hat{0}_n^\dagger\hat{e}_n \text{ and } \hat{C}_n = \sqrt{\Gamma_0}\ \hat{e}_n^\dagger\hat{0}_n, \tag{35}$$

the internal state $|0\rangle$ standing for the $1^1\mathrm{S}_0$ state and $\Gamma_0 = 1/\tau$. Since we are not interested in collective effects due to the decay processes, we modelled them as conserving the motional state while flipping the internal state from $|\mathrm{e}\rangle$ to $|0\rangle$. The solution $\rho(t)$ is numerically calculated using either an exact solving method [4, 5] or a quantum Monte-Carlo one [6]. From the density matrix, the population of each manifold, $|\mathrm{g}\rangle$, $|\mathrm{e}\rangle$, or $|0\rangle$, is extracted according to:

$$\langle n_i(t)\rangle = \sum_n \mathrm{Tr}\left[\hat{i}_n^\dagger\hat{i}_n\rho(t)\right]/N, \tag{36}$$

i standing for one of these three states and N being the total number of particles. Due to the computational time increasing very fast, we limited our analysis to few-atoms systems not exceeding $N = 3$.

A first thing that we confirmed is that no many-body effects can be observed if only carrier transitions are allowed and the Fermi gas can be treated as an ensemble of independent particles. In contrast, the dynamics is modified by the presence of the sideband couplings enabling cross-talk between the particles. The coherent dynamics exhibits Pauli blocking and can not be obtained by considering independent single-particles. Supplementary Figure 1 shows an example of such behaviour for different numbers of fermions in the system. Having more than a single atom results in an enhanced lower state depletion due to Pauli blockade.

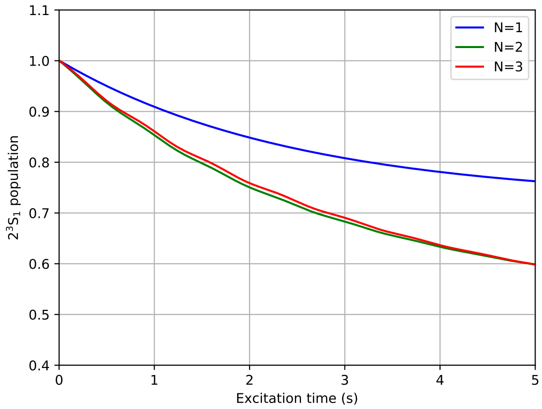

Supplementary Figure 1. Evolution of the population in the $2^3\mathrm{S}_1$ state upon excitation to the $2^1\mathrm{S}_0$ state for a system composed of 1 to 3 fermions obtained by solving the Master equation. In this example, the initial motional states of the atoms are centered around $n = 100$, in the energy range where the Pauli blockade changes the dynamics of the system.

We then simulated line profiles by modelling the Fermi gas as an ensemble of sets of N particles each of which is weighted by the Fermi-Dirac distribution. The remaining $|g\rangle$ population of each of those is calculated and added to obtain the total remaining population. The procedure is repeated for different detunings to simulate a line profile. Supplementary Figure 2.a. shows the results of these simulations with increasing N from 1 to 3. In our experiment, the Fermi gases are composed of $10^5 - 10^6$ atoms, hence we are only interested in a trend for the linewidth as it is not possible to simulate the full system. The excitation strength increases

with N and the profile narrows as shown in Supplementary Figure 2.b., displaying a similar behaviour as what we experimentally observe.

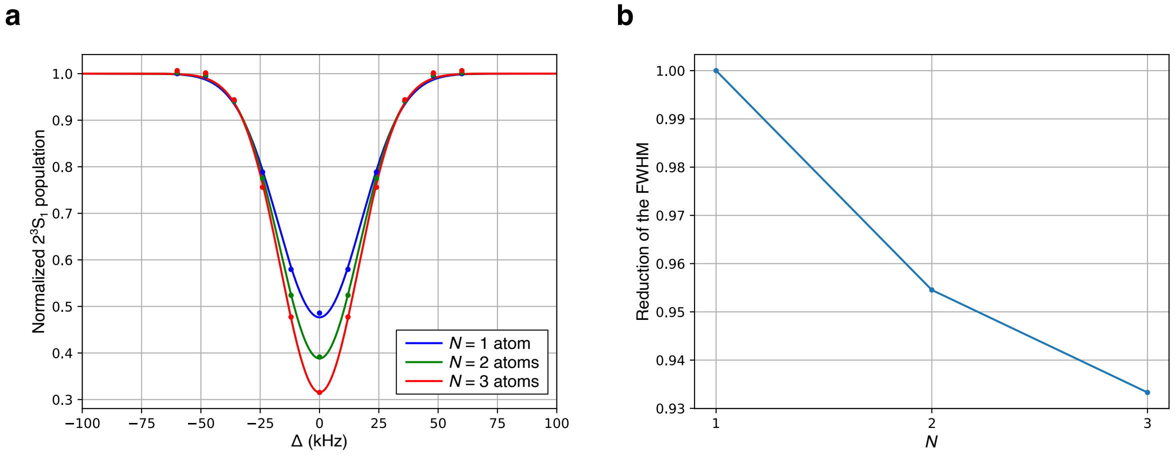

Supplementary Figure 2. a. Simulation of line profiles with sets of nearest energy neighbours ranging from 1 to 3 atoms. b. Extracted FWHM as a function of the number of atoms. Increasing the number of fermions results in a decrease of the FWHM.

Supplementary Note 4: Averaging of the Rabi frequency

In order to estimate the average Rabi frequency that we use for solving the four level dynamics explained in the main text, we follow a similar approach as in Ref.[7]. We label the states with two indices, $n_{/\!/}$ and $n_\perp$, which represent the axial and radial directions respectively. With this notation, excitation light of angular frequency $\omega = \omega_0 + \omega_{\mathrm{rec}} + \ell\omega_{/\!/}$ couples only the sets of internal (electronic) and motional states $|2^3\mathrm{S}_1\rangle|n_{/\!/}\rangle$ and $|2^1\mathrm{S}_0\rangle|n_{/\!/}+\ell\rangle$ with a Rabi frequency $\Omega_{n_{/\!/},n_{/\!/}+\ell}$ given by the equation (2). The averaged Rabi frequency is thus given by:

$$\langle\Omega\rangle = \frac{1}{N}\sum_{n_{/\!/},n_\perp} f_{\mathrm{FD}}(n_{/\!/},n_\perp)\;\Omega_{n_{/\!/},n_{/\!/}+\ell}, \tag{37}$$

with the normalization $N = \sum_{n_{/\!/},n_\perp} f_{\mathrm{FD}}(n_{/\!/},n_\perp)$, and the Fermi-Dirac distribution given by:

$$f_{\mathrm{FD}}(n_{/\!/},n_\perp) = \frac{1}{1+e^{-\beta\mu+\beta\hbar n_{/\!/}\omega_{/\!/}+\beta\hbar n_\perp\omega_\perp}}. \tag{38}$$

Supplementary Note 5: Evaluation of the effective saturation intensity

In addition to the estimation of the broadening induced by the presence of the depumper laser, the results from the numerical solving of the optical Bloch equations described in the Methods section are also used to determine the effective saturation intensity of the system. We are interested in the effect of the depumper laser (which pumps atoms from the $2^1\mathrm{S}_0$ to the $4^1\mathrm{P}_1$ states) onto the remaining population in the $2^3\mathrm{S}_1$ state which constitutes our observable. Even though the $2^1\mathrm{S}_0$ and $4^1\mathrm{P}_1$ states taken alone would suggest a saturation intensity of about $2.3\,\mathrm{mW/cm^2}$, the evolution of the $2^3\mathrm{S}_1$ population is highly dependent on the dynamics of the intermediate $2^1\mathrm{S}_0$ state. The presence of the depumper light thus results in a reduction of the lifetime of this state, broadening it such that the atoms are ultimately prevented from leaving the $2^3\mathrm{S}_1$ state when the depumper intensity is sufficient. This regime defines the relevant effective saturation intensity for this cascadated system.

The remaining population in the $2^3\mathrm{S}_1$ state is evaluated as a function of the depumper intensity, I. Even though the level scheme is not a simple two-level system, the obtained curve shows a clear saturation effect which is fitted with the following elementary saturation function:

$$n_{\mathrm{triplet}}(I) = 1 - \frac{1}{1+I/I_{\mathrm{sat}}}, \tag{39}$$

allowing to extract an effective saturation intensity $I_{\mathrm{sat}} \simeq 14.7\,\mu\mathrm{W/cm^2}$, as shown in Supplementary Figure 3. The system composed of the combination of the $2^3\mathrm{S}_1 \to 2^1\mathrm{S}_0$ and $2^1\mathrm{S}_0 \to 4^1\mathrm{P}_1$ transitions thus saturates for a much lower intensity than this last one alone.

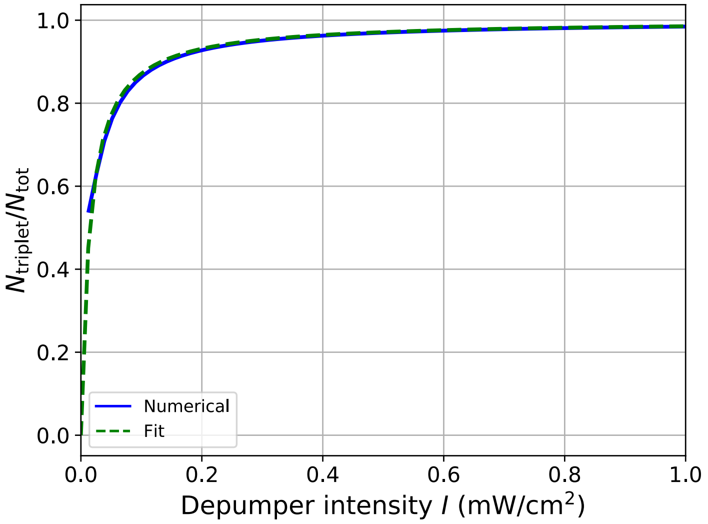

Supplementary Figure 3. Remaining fraction of the population in the $2^3\mathrm{S}_1$ state versus intensity of the depumper. The results obtained from numerically solving the optical Bloch equations are shown as a blue solid line, while the result of a fit to the function (39) is shown as a green dashed line.

Supplementary Note 6: Comparison of the behaviour of different datasets

As explained in the main text, we varied the intensity of the depumper beam for Fermi gases with different thermodynamical parameters. To achieve totally different T/T_{F} values, we changed the intensity of the ODT beams. All the datasets we gathered this way exhibit the same behaviour as the one shown in the main text. Supplementary Figure 4 shows a summary of two additional datasets obtained with different trap depth than Figure 3 of the main text. The intensity value for which the transition between the two regimes happens is changed (also compared to the dataset shown in the main text) because of the interplay between the laser linewidth, the effective lifetime due to the pump to the $4^1\mathrm{P}_1$ state and the trap frequency. We did not take these data into account in the main text because it corresponds to an early implementation of the experimental procedure and the parameters were not well-controlled.

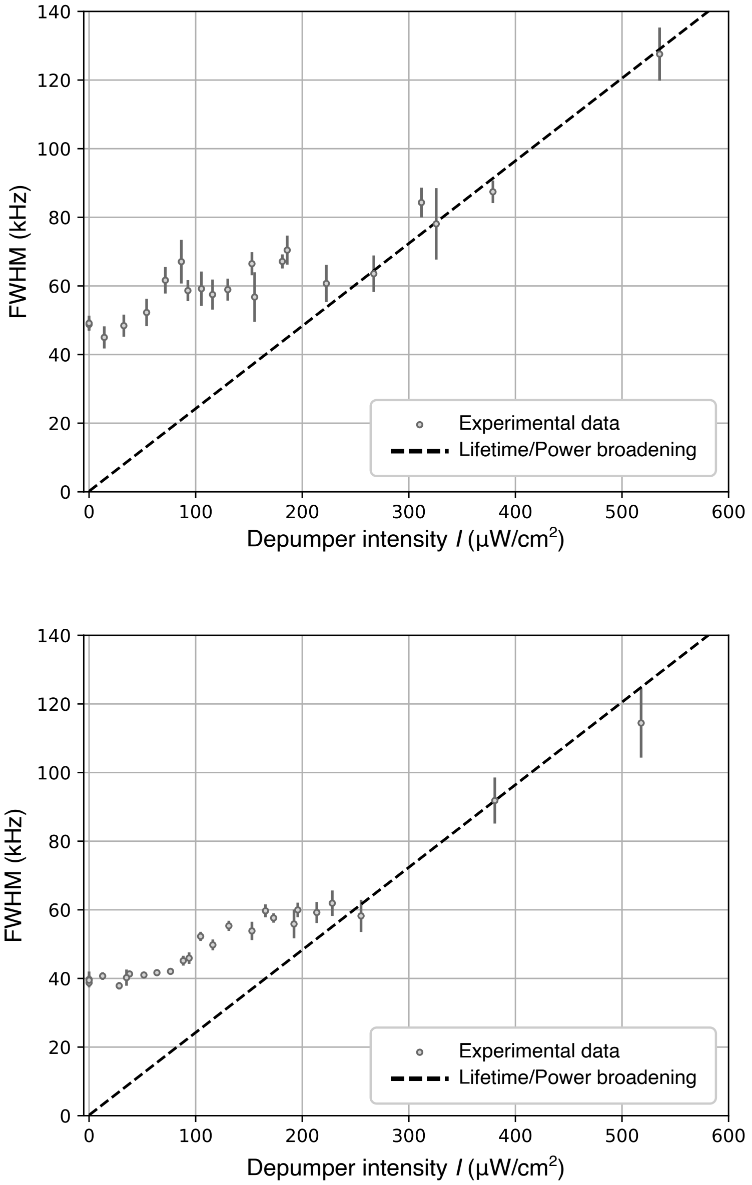

Supplementary Figure 4. Variation of the FWHM as a function of depumper laser intensity for deeper dipole traps than the data shown in the main text. The errorbars on these points represent the error on the FWHM obtained from fitting Gaussian profiles to the measured line profiles. Each of the two datasets were acquired with similar DFGs composed of 10^6 atoms at a temperature of 300 nK and exhibit the same behaviour as the dataset shown in the main text.

Supplementary References

[1] D. Leibfried, R. Blatt, C. Monroe, and D. Wineland, *Quantum dynamics of single trapped ions*, Rev. Mod. Phys. **75**, 281 (2003)

[2] G. Juzeliunas, and M. Masalas, *Absorption by Cold Fermi Atoms in a Harmonic Trap*, Phys. Rev. A **63**, 061602 (2001)

[3] R.P.M.J.W. Notermans, R.J. Rengelink, and W. Vassen, *Comparison of Spectral Linewidths for Quantum Degenerate Bosons and Fermions*, Phys. Rev. Lett. **117**, 213001 (2016)

[4] S. Machnes, and M. Plenio, *Surprising Interactions of Markovian noise and Coherent Driving*, arXiv:1408.3056 (2014)

[5] M. Am-Shallem, A. Levy, I. Schaefer, and R. Kosloff, *Three approaches for representing Lindblad dynamics by a matrix-vector notation*, arXiv:1510.08634v2 (2015)

[6] K. Mølmer, Y. Castin, and J. Dalibard, *Monte Carlo wave-function method in quantum optics*, J. Opt. Soc. Am. B **3**, 524-538 (1993)

[7] G. K. Campbell, M. M. Boyd, J. W. Thomsen, M. J. Martin, S. Blatt, M. D. Swallows, T. L. Nicholson, T. Fortier, C. W.Oates, S. A. Diddams, N. D. Lemke, P. Naidon, P. Julienne, Jun Ye, A. D. Ludlow, *Probing Interactions between ultracold fermions*, Science **324**, 5925, 360-363 (2009)
